# Incorporation of Immune Checkpoint Blockade into Chimeric Antigen Receptor T Cells (CAR-Ts): Combination or Built-In CAR-T

**DOI:** 10.3390/ijms19020340

**Published:** 2018-01-24

**Authors:** Dok Hyun Yoon, Mark J. Osborn, Jakub Tolar, Chong Jai Kim

**Affiliations:** 1 Department of Oncology, Asan Medical Center, University of Ulsan College of Medicine, Seoul 05505, Korea; dhyoon@amc.seoul.kr; 2 Asan-Minnesota Institute for Innovating Transplantation, Asan Institute for Life Sciences, Asan Medical Center, University of Ulsan College of Medicine, Seoul 05505, Korea; ckim@amc.seoul.kr; 3 Asan-Minnesota Institute for Innovating Transplantation, University of Minnesota, Minneapolis, MN 55455, USA; tolar003@umn.edu; 4 Department of Pediatrics, University of Minnesota Medical School, Division of Blood and Marrow Transplantation, Minneapolis, MN 55455, USA; 5 Masonic Cancer Center, University of Minnesota, Minneapolis, MN 55455, USA; 6 Stem Cell Institute, University of Minnesota, Minneapolis, MN 55455, USA; 7 Center for Genome Engineering, University of Minnesota, Minneapolis, MN 55455, USA

**Keywords:** adoptive T cell therapy, chimeric antigen receptors, PD-1, immune-checkpoint, cancer immunotherapy, gene editing, gene therapy, CRISPR/Cas9

## Abstract

Chimeric antigen receptor (CAR) T cell therapy represents the first U.S. Food and Drug Administration approved gene therapy and these engineered cells function with unprecedented efficacy in the treatment of refractory CD19 positive hematologic malignancies. CAR translation to solid tumors is also being actively investigated; however, efficacy to date has been variable due to tumor-evolved mechanisms that inhibit local immune cell activity. To bolster the potency of CAR-T cells, modulation of the immunosuppressive tumor microenvironment with immune-checkpoint blockade is a promising strategy. The impact of this approach on hematological malignancies is in its infancy, and in this review we discuss CAR-T cells and their synergy with immune-checkpoint blockade.

## 1. Introduction

**Figure 1:**
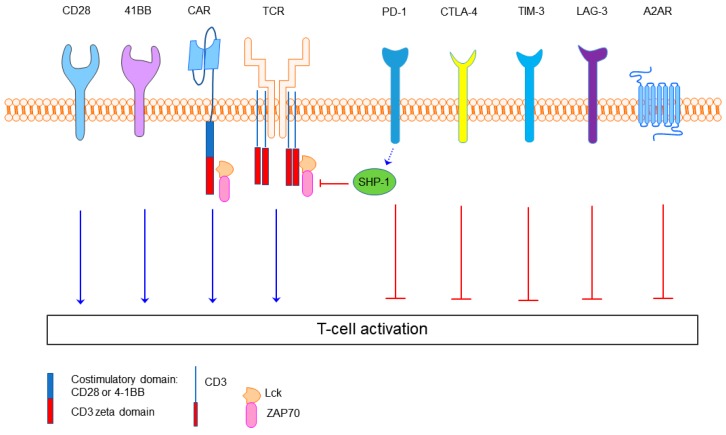
The structure of the second-generation chimeric antigen receptor (CAR) and examples of the immune checkpoints. The second-generation CAR is composed of a single-chain Fv domain (scFv) targeting tumor cells, hinge and transmembrane domains along with the CD28 or 4-1BB, and CD3 zeta co-stimulatory domains. T cell receptor (TCR) kinases like Lck and ZAP70 relay the activation signal of TCR and CAR once antigen dependent receptor clustering has occurred. Inhibitory molecules include Src homology 2 domain-containing protein tyrosine phosphatase 1 (SHP-1) PD-1, CTLA-4, TIM-3, LAG-3 and adenosine 2A receptor (A2AR). SHP-1 is an inhibitory protein tyrosine phosphatase that can dephosphorylate the intracellular domains. PD-1 is a pivotal immune checkpoint receptor involved in T cell exhaustion. CTLA-4 is an immune checkpoint that competes with CD28 for its B7 ligands. TIM-3 is another immune checkpoint which is highly expressed in exhausted T cells.

The immune system plays a crucial role in controlling malignancies. Nevertheless, tumors can evade the immune system with multiple mechanisms including downregulation of major histocompatibility complex (MHC) or upregulating immunoinhibitory molecules. These so called immune checkpoints normally serve as a brake on immune cell overactivity and prevent autoimmune reactivity. Tumor acquisition of these properties leads to tumor cell evasion and progression [1]. The programmed cell death-1 receptor (PD-1, CD279) axis has been recognized as a pivotal immune checkpoint. PD-1 on activated T cells with its ligands PD-L1 (B7-H1 or CD274) and PD-L2 (B7-DC or CD273) maintains immunologic tolerance through the suppression of auto-reactive T cells [2]. Likewise, in the tumor microenvironment, the interaction of the PD-1 on tumor infiltrating T cells (TILs) with its ligands, PD-L1 and/or PD-L2 on the surface of antigen presenting cells and malignant cells inhibits TIL potency (Figure 1). Immune checkpoint blockade, including anti-PD-1/PD-L1 and anti-CTLA-4, has led to a breakthrough in the treatment of multiple types of advanced solid tumors by preventing checkpoint molecule triggered exhaustion. It therefore represents a powerful weapon in the anti-tumor treatment arsenal. 

In hematological malignancies, immune-checkpoint blockade is associated with rather modest effects, save for relapsed or refractory Hodgkin lymphoma. In this class of tumors a highly effective approach has been the use of chimeric antigen receptor (CAR)-T cells. In this review we discuss CAR-T cells and their potential synergy with immune-checkpoint blockade in treating malignancy.

CAR-T cells are the culmination of a long history of adoptive cellular immunotherapy and genetic engineering. This engineering approach redirects a patient’s own T cells to kill target tumor cells. This occurs via the CAR molecule that is composed of a single-chain Fv antigen recognition domain (scFv), a hinge, a transmembrane domain, co-stimulatory domain(s), and the T cell receptor CD3 zeta chains. Ectopic expression of this CAR in T cells enables antigen recognition and engagement through the scFv triggering T cell activation. The US Food and Drug Administration (FDA) has approved two CD19-specific CAR-T cell therapies for B-cell malignancies in 2017: tisagenlecleucel (CTL019) for children and young adults with B-cell acute lymphoblastic leukemia (B-ALL) that is refractory or has relapsed at least twice, and axicabtagene ciloleucel (KTE-C19) for the treatment of adult patients with relapsed or refractory diffuse large B-cell lymphoma (DLBCL). Despite some stunning therapeutic successes, CAR-T cells, similar to endogenous T cells, can also demonstrate susceptibility to inhibitory immune checkpoints present in the tumor microenvironment. 

## 2. Current Status of CAR-T

Approval of tisagenlecleucel for relapsed or refractory pediatric B-ALL was based on the promising outcomes from ELIANA trial, the first global multicenter trial of a CAR-T cell therapy, with a confirmed overall remission rate of 82.5% with a relapse-free probability of 75% at 6 months and 64% at 12 months among responders [3]. Of data from other trials, the most compelling showed that CD19-specific CAR-T cells can achieve complete remission (CR) rates of 70–94% in refractory B-ALL [1,2,3,4]. The use of axicabtagene ciloleucel, the other FDA-approved CD19-specific CAR-T, resulted in 52% patients with relapsed or refractory DLBCL achieving CR in a multicenter clinical trial (ZUMA-1) [4]. Other multicenter trials for DLBCL also showed encouraging preliminary data, with CR rates ranging from 43% to 52% [5,6,7]. This efficacy is remarkable, as only 8% could achieve CR with a median overall survival of 6.6 months after being treated with conventional therapies [7]. However, the CR rate in CAR-T cell use in chronic lymphocytic leukemia (CLL) diminishes to 29% in a trend that extends to other/solid tumors [8,9,10,11,12,13]. For example, there were only two responders and 5 patients with stable disease (SD) out of 11 in a phase I study of epidermal growth factor receptor (EGFR)-directed CAR-T cells for relapsed/refractory EGFR^+^ non-small cell lung cancer patients [9]. A dose-escalation phase I/II study established the feasibility and safety of administering HER2-targeted CAR-T cells to patients with recurrent or refractory HER2-positive tumors; however, the clinical benefit was limited, as only 4/17 evaluable patients exhibited SD [12].

The reason for this difference between tumors is being actively explored, and is likely multifactorial. One primary reason for this disparity is the tumor-intrinsic mechanisms and the associated tumor microenvironment that play an important role in the inhibition of the antitumor immune response [14]. It is well established that solid malignancies create an environment that can impede T cell activity. This includes the presence of regulatory T cells (Tregs), tumor-associated macrophages (TAM), myeloid-derived suppressor cells (MDSC), and cancer-associated fibroblasts, that promote higher levels of inhibitory ligands and cytokines. These phenomena appear to extend in part to liquid tumors as evidenced by elevated numbers of Tregs in CLL and DLBCL [14,15]. Further, tumor indoleamine 2,3-dioxygenase (IDO) is known to inhibit CD19-CAR-T cells in a xenograft lymphoma model expressing IDO [15]. On a molecular level, numerous inhibitory immune checkpoint molecules are upregulated. Accordingly, attempts have been made to modulate this pathway in order to promote better CAR-T cell activity in the tumor environment.

## 3. Rationale for Incorporating PD-1 Blockade into CAR-T

### 3.1. PD-1 and Cancer

PD-1 is a crucial molecule that suppresses the immune response [16]. Chronic antigenic stimulation can lead to progressive phenotypic and functional changes, called “T cell exhaustion”. This includes the loss of proliferative capacity from and by interleukin-2 (IL-2). Tumor necrosis factor α (TNF-α) and interferon gamma (IFN-γ) production is diminished, which generally coincides with expression of inhibitory surface receptors such as PD-1, LAG-3, CD160, 2B4, TIM-3, and CTLA-4 [17,18]. PD-L1 is a PD-1 ligand that plays an important role in the inhibition of T cell-mediated immune responses. Tumors can escape host immune surveillance by expressing PD-L1 [19]. It is expressed in various solid malignancies, including squamous cell carcinoma of the head and neck, melanoma, and lung cancers [20]. Tumors also exhibit upregulation of PD-L1 when being targeted by T-cells, mainly in response to IFN-γ [21]. CAR-T cells also exhibited upregulation of immune checkpoint molecules including: CTLA-4, PD-L1, and LAG-3. These markers have been observed within the tumor microenvironment in clinical samples from the ZUMA-1 trial [22]. PD-L1 is expressed not only in solid tumors but also in non-Hodgkin lymphoma including DLBCL, and is known to inhibit the activity of tumor-associated T cells [23,24]. Its expression is an independent indicator of poor prognosis [21,25]. In particular, PD-L1 protein expression is associated with activated B-cell-like or non-germinal center B cell-like phenotypes of DLBCL, which are poor prognostic subgroups [25,26]. Blockade of the PD-1/PD-L1 axis has led to meaningful responses in B-cell non-Hodgkin lymphomas with an overall response rate of 36% for DLBCL and 40% for follicular lymphoma (FL) [16,27,28].

### 3.2. PD-1 or PD-L1 Blockade

Blocking antibodies against PD-1/PD-L1, such as nivolumab, pembrolizumab, or atezolizumab, are under active clinical development. Beneficial activity in many malignancies has been demonstrated and these antibodies have become standard in the treatment of several types of solid tumors, including melanoma, non-small cell lung cancer, renal cell carcinoma, and head and neck carcinoma [29,30,31]. CLL, another malignancy with lower response rates to CAR-T cells, is known to have a tumor-supportive microenvironment with depressed immune cell function. Exhausted T cells, defective immunologic synapse formation, and immunosuppressive myeloid cells are observed. Aberrant expression of PD-L1 on CLL cells and MDSCs from the peripheral blood of CLL patients is also prevalent [18,32,33]. PD-L1 blockade has been shown to normalize CD4:CD8 ratios and restore CD8 T cell cytotoxicity and immune synapse ex vivo and in vivo [18].

### 3.3. Rationale for Employing PD-1 Blockade for CAR-T on CAR-T Side: Upregulation of Immune Checkpoints in CAR-T Cells

Like their endogenous counterparts, CAR-T cells also acquire a differentiated and exhausted phenotype associated with increased expression of PD-1 [34,35,36,37]. A significant fraction of both CD28 and 4-1BB mesothelin-specific CAR-T cells were found to co-express PD-1 and LAG-3 or PD-1 and TIM-3 in an orthotopic mouse model of pleural mesothelioma [38]. In another model of human mesothelin-expressing flank tumors, mesothelin-specific CAR-TILs rapidly lost functional activity, with the expression of surface inhibitory receptors of PD-1, LAG-3, TIM-3, and 2B4 and upregulation of intrinsic T cell inhibitory enzymes (diacylglycerol kinase and SHP-1) [39]. This hypofunction was reversed when the T cells were removed from the tumor. Addition of an anti-PD-L1 antibody could significantly restore the killing activity and the ability to secrete IFN-γ by mesothelin-specific CAR-TILs ex vivo. Third-generation GD2-specific CAR-T cells showed reduced cytokine production after long-term culture with melanoma cell lines that was reversed by anti-PD-1 [40]. Significant activation-induced cell death of CAR-T cells was observed after repeated antigen stimulation, which was PD-1 dependent and PD-1 blockade could promote killing of PD-L1^+^ tumor cell lines and enhanced CAR-T cell survival in vitro.

An increase in the expression of these inhibitory surface receptors has also been directly confirmed in clinical samples. In 8 of 11 lymphoma patients infused with CD19-specific CAR-T cells, PD-1 expression increased on CD4^+^ CAR-positive cells by at least three-fold from the time of infusion to the time of peak blood levels [34]. In a trial involving patients with B-cell malignancies that had progressed after allogeneic stem cell transplantation received a single infusion of CD19-specific CAR-T cells, the fraction of both CD8^+^ and CD4^+^ CAR-T cells expressing PD-1 significantly increased between the time of infusion and post adoptive cell transfer (ACT) analysis (*p* < 0.001 for both CD8^+^ and CD4^+^ CAR-T cells). PD-1 expression was also higher on CAR-T cells than non-CAR-T cells [35]. In addition, GD2-specific CAR-T cells demonstrated upregulation of PD-1 and PD-L1 and limited persistence in patients with metastatic melanoma enrolled in a phase 1 clinical [40].

## 4. Combination of CAR-T and Anti-PD-1 Antibody

### 4.1. Combination of PD-1 Blockade in Preclinical Models: Anti-PD-1 or PD-L1 Antibodies Can Boost CAR-T Cell Therapy In Vivo

**Figure 2:**
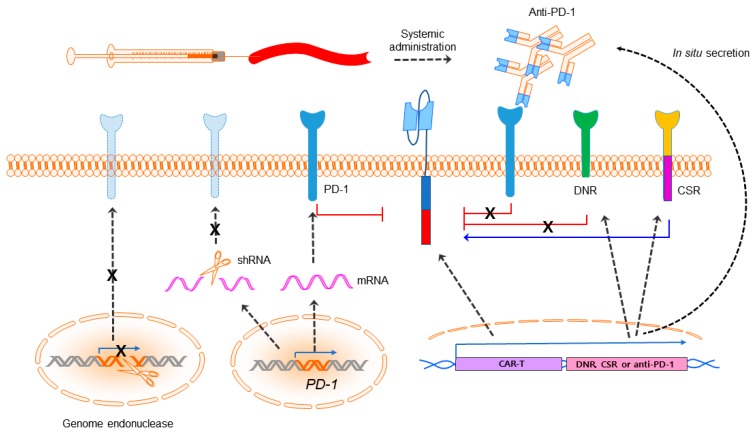
Immune checkpoint blockade. CAR-T cells can be augmented in efficacy with PD-1 blockade by systemic combination of anti-PD-1 or anti-PD-L1 antibodies and being engineered to secrete anti-PD-1/PD-L1 by CAR-T cells or express a PD-1 dominant negative receptor (DNR) or a PD-1:CD28 chimeric switch-receptor (CSR). Expression of PD-1 also can be downregulated by a PD-1 shRNA lentiviral cassette or PD-1 deficient CAR-T can be generated utilizing programmable genome editing endonucleases. The black dashed arrow indicates expression of the genes unless specified. The signs “X” denotes steps prohibited.

Results of preclinical experiments in numerous mouse models have demonstrated that combining CAR-T cell therapy with PD-1 pathway blockade can improve CAR-T cell activity and promote in increased tumor cell death (Figure 2) [38,41]. John et al. first showed that the administration of a PD-1 blocking antibody could increase the therapeutic activity of CAR-T cells against HER2^+^ tumors (Table S1) [42]. They observed a significant increase in the level of PD-1 expression on transduced HER2-specific CD8^+^ CAR-T cells following antigen-specific stimulation. Further, markers of activation and proliferation were increased in CAR-T cells in the presence of anti-PD-1 antibody. In ACT studies, they showed a significant improvement in growth inhibition of HER2^+^ tumors treated with CAR-T cells in combination with an anti-PD-1 antibody. Strikingly, a decrease in the percentage of MDSCs was also observed in the tumor microenvironment of mice treated with a combination treatment of CAR-T and anti-PD-1 antibody. Moreover, Cherkassky et al. showed that PD-1/PD-L1 blockade can restore the effector function of CD28 mesothelin-specific CAR-T cells using an orthotopic mouse model of pleural mesothelioma [38]. In addition, Moon et al. showed that anti-NY-ESO-1 T cell receptor (TCR)-engineered T cells became severely hypofunctional and were accompanied by upregulation of PD-1, TIM-3, and LAG-3 in a high percentage of cells [43]. Repeated intraperitoneal injections of anti-human PD-1 antibody augmented the efficiency of adoptively transferred anti-NY-ESO-1 TCR-engineered T cells in controlling the growth of tumors, and preserved TIL function. In a liver metastasis model expressing carcinoembryonic antigen (CEA), Burga et al. showed that in MDSC, PD-L1 suppressed antitumor responses through engagement of PD-1 on CD28 CEA-specific CAR-T cells [44]. Granulocyte-macrophage colony-stimulating factor (GM-CSF), in cooperation with STAT3, promoted PD-L1 expression in MDSC. CAR-T efficacy was rescued when mice received CAR-T in combination with MDSC depletion, GM-CSF neutralization to prevent MDSC expansion, or PD-L1 blockade with anti-PD-L1 antibody. Collectively, these xenogeneic models provided impetus for human studies.

However, it is notable that while a high-dosage (250 μg/mouse of anti-PD-1 antibody) PD-1 blockade was capable of enhancing the antitumor activity of anti-HER2 CAR-T cells in a syngeneic breast cancer model [42], the antibody failed to inhibit tumor growth or enhance the antitumor efficacy of CAR-T cells at a low dose (125 μg/mouse) [45]. In addition, multiple doses of PD-1 blocking antibodies have been required to rescue T cell activity [14,46]. These results suggest that optimal doses and schedules of PD-1 blockade will be required in order to maximize the synergy of the individual agents.

### 4.2. Clinical Evidence on the Combination of PD-1 Blockade and CAR-T Cells

Clinical experience employing the combination of CAR-T and immune checkpoint blockade is in its early stages; however, encouraging data are emerging. Six pediatric B-ALL patients were treated with pembrolizumab to augment response to CD19-specific CAR-T cells and three patients showed clinical responses with prolonged persistence of CAR-T cells [46]. Interestingly, the three responders all received pembrolizumab continuously every 3 weeks while the other nonresponding patients received just a single dose. A patient treated with CAR-T cells for the first time after relapse was treated with pembrolizumab following signs of tumor progression, which resulted in increased CAR-T cells in the peripheral blood and decreased tumor burden demonstrated by positron emission tomography (PET). Off-tumor side effects were limited to two cases of fever, two cases of cytopenia, and there was no incidence of severe cytokine release syndrome (CRS). In addition, a response to PD-1 blockade with pembrolizumab was observed in a patient with primary mediastinal large B-cell lymphoma refractory to CD19-specific CAR-T cell therapy [47]. Pembrolizumab was initiated on day 26 following CAR-T cell infusion in a 35-year-old man showing progressive disease after CAR-T infusion and high PD-L1 expression in the tumor. A chest computed tomography (CT) performed 3 weeks after pembrolizumab delivery showed improvement in mediastinal and pulmonary lesions that continued for 12 months and was accompanied by an expansion of CAR-T cells with decreased coexpression of PD-1 by CAR-T cells. Based on these findings, a clinical trial is underway in which patients with refractory or relapsed DLBCL, FL, or mantle cell lymphoma following CD19-specific CAR-T cell infusion are treated with pembrolizumab in an attempt to reactivate exhausted CAR-T cells (NCT02650999; Table 1). In addition, multiple clinical trials investigating the combination of CD19-specific CAR-T cells and PD-1 or PD-L1 blockade are ongoing (Table 1).

While these findings suggest that immune-checkpoint blockade might restore or enhance the anticancer activity of CAR-T cells, a small phase 1 trial in a solid tumor did not find significant benefit of PD-1 blockade. Heczey et al. [49] reported data from a third-generation GD2-CAR for relapsed or refractory neuroblastoma patients in three cohorts: Cohort 1 receiving CAR-T alone, cohort 2 receiving CAR-Ts plus cyclophosphamide and fludarabine (Cy/Flu), and cohort 3 receiving CAR-Ts, Cy/Flu, and pembrolizumab. Two doses of pembrolizumab were administered to patients in cohort 3 on days −1 and +21 of CAR-T cell infusion. Antitumor responses at 6 weeks were modest, with no difference among the cohorts. Unlike the above experience in B-cell malignancies, pembrolizumab had no measurable effect on CAR-T cell expansion, persistence, or circulating cytokine levels. However, it is interesting that only two patients with SD in cohort 3 could achieve and maintain CR after salvage treatment. Collectively, the use of PD-1 blockade are promising when paired with CAR-T cells; however, each tumor class will require rigorous definition of the optimal conditions to achieve maximal responses. In addition, careful attention is mandated for the potential toxicities such as CRS or neurologic damage that may occur in combinatorial strategies due to T cell overactivation.

## 5. Built-In CAR-T; Incorporation of PD-1 Blockade into CAR-T Cells

### 5.1. In Situ Secretion of Anti-PD-1 or Anti-PD-L1 Antibodies

Suarez et al. developed a combinatorial immunotherapy that consisted of human CD28 anti-carbonic anhydrase IX (CAIX)-targeted CAR-T cells engineered to secrete human anti-PD-L1 antibodies at the tumor site using a single bicistronic lentiviral vector [50]. This local anti-PD-L1 antibody delivery led to a five-fold reduction in tumor growth and a 50–80% reduction in tumor weight when compared with anti-CAIX CAR-T cells alone in a humanized mice model of clear cell renal cell carcinoma. In addition, Li et al. developed engineered CD28 CD19-specific CAR-T cells secreting human anti-PD-1 antibody and demonstrated that the secretion of anti-PD-1 can enhance the antitumor activity of CAR-T cells and prolonged overall survival in a xenograft mouse model of CD19 expressing solid tumor [45]. Interestingly, the xenograft tumor model treated with a low dose of anti-PD-1 antibody failed to inhibit tumor growth or enhance the antitumor efficacy of CAR-T cells even though the amount of circulating antibody (~0.7 mg/mL) was still 15-fold higher than the amount detected in the anti-PD-1 antibody secreting CAR-T cell treatment group. In addition, they found that anti-PD-1 antibody secreting CAR-T cells had a more functional phenotype with a higher proliferation potential without exhaustion than parental CAR-T cells at the local tumor site [45].

Tanoue et al. developed a new immunotherapy strategy for the treatment of prostate cancer that used a combination of intratumoral injection of oncolytic adenovirus (Onc.Ad) and a helper-dependent adenovirus (HDAd) that expressed a PD-L1 blocking mini-antibody along with intravenous CD28 HER2-specific CAR-T cells [51]. They demonstrated that coadministration of CAR-T cells and Onc.Ad/HDAd could enhance the antitumor effects in relation to treatment with either CAR-T cells alone or with CAR-T cells plus Onc.Ad. Tumor cells expressed 50% less PD-L1 and CAR-T cells showed 30% lower PD-1 expression compared to other groups. In addition, the benefits of locally produced PD-L1 mini-body was superior to the infusion of anti-PD-L1 IgG. Some mice that received systemic infusion of anti-PD-L1 IgG exhibited transient diarrhea; however, no severe immune-related side effects were noted in mice treated with CAR-T and Onc.Ad/HDAd.

In sum, these “built-in” therapies show promise and CAR-T cells that secrete either anti-PD-1, anti-PD-L1 or anti-CTLA-4 antibodies are currently being evaluated in clinical trials (Table 2).

### 5.2. Engineering of PD-1: Dominant-Negative Receptor or Chimeric Switch-Receptor

Cherkassky et al. also studied the effect of cell intrinsic PD-1 signaling blockade by incorporating a PD-1 dominant-negative receptor (DNR) [38]. This molecule contains the extracellular ligand-binding domain of the receptor fused to a CD8 transmembrane domain in order to compete for PD-1 ligand binding. CD28 CD19-specific CAR-T cells co-transduced with PD-1 DNR demonstrated enhanced in vitro T cell functions and in vivo T cell efficacy with enhanced tumor burden control and prolonged median survival.

When PD-1 was fused to a T cell co-stimulatory receptor by substituting its transmembrane and intracellular domains with the CD28 domain, adoptively transferred T cells modified to express this PD-1:CD28 chimeric switch-receptor (CSR) exhibited enhanced functionality without exhaustion upon engagement of PD-L1^+^ tumors [52]. Liu et al. employed this approach to overcome the PD-L1 immunosuppressive effects on CAR-T cells [53]. Inhibitory PD-L1/PD-1 signaling was blocked while providing CD28 costimulation led to augmented antitumor efficacy with decreased susceptibility to tumor-induced hypofunction.

### 5.3. Knockdown or Knockout of PD-1: shRNA or Clustered Regularly Interspaced Short Palindromic Repeats (CRISPR)/Cas9

Cherkassky et al. also tested CD28 mesothelin-specific CAR-T cells expressing PD-1 targeting shRNAs, which showed enhanced proliferative function upon antigen stimulation, augmented cytotoxicity, and enhanced cytokine secretion [38]. There is an active clinical trial of CD19-specific CAR with a PD-1 shRNA lentiviral cassette for CD19 positive B-cell lymphoma (NCT03208556). However, it is noteworthy that these PD-1 shRNA–transduced CAR-T cells did not achieve greater in vivo tumor rejection, despite more than 60% PD-1 receptor knockdown efficiency [38]. In light of these results and with the rapid proliferation of programmable nucleases that can be designed, built, and tested in short order the merging of CAR-T with genome engineering is highly promising.

Unlike RNAi-mediated knockdown, in which gene function is reduced, genome editing can target a nuclease to a user defined sequence where it cleaves the DNA helix. Following this double stranded DNA break the cell repairs the lesion via the error prone non-homologous end joining (NHEJ) pathway. This results in small insertions and deletions that can permanently disrupt the genetic code, causing a knockout with elimination of gene function in modified cells. Rupp et al. developed a protocol to generate PD-1-deficient CD19-specific CAR-T cells utilizing CRISPR/Cas9-mediated PD-1 disruption [54]. They routinely observed a >50% reduction of PD-1^+^ CAR-T cells 48 hours post-editing, and PD-1 could be successfully ablated in both CD4^+^ and CD8^+^ cells. PD-1 disruption potentiated CAR-T cell-mediated killing of tumor cells in vitro and enhanced clearance of PD-L1^+^ tumor xenografts in vivo. Ren et al. also tested whether disruption of PD-1 by CRISPR/Cas9 in CAR-T cells would enhance antitumor activity [55]. The proportion of PD-1^−^ CAR-T increased to 59.1% compared with 8.1% in control CAR-T cells, while prostate specific cancer antigen (PSCA)-specific PD-1^−^ CAR-T cells showed significantly enhanced antitumor activity compared with conventional CAR-T cells. These studies demonstrate improved therapeutic efficacy of CRISPR/Cas9-edited CAR-T cells and highlights the potential of precision genome engineering to enhance next-generation cell therapies. A clinical trial of PD-1 knockout engineered CD19-speicific CAR-T is currently being employed (NCT03298828).

## 6. Next Generation Therapies. Ablation of Auto-Reactive TCRs by Genome Editing in Combination with Checkpoint Blockade

### 6.1. Knockout of TCR, Beta2-Microglobulin and/or Human Leukocyte Antigen (HLA)

A potential caveat in approaches to augment CAR-T efficacy by PD-1 blockade is that CAR-T cells might also express auto-reactive TCRs, resulting in autoimmune side effects similar to those observed with systemic PD-1 antibody blockade [56]. As a solution to TCR-driven host tissue reactivity, gene-editing nucleases have been employed to disrupt components of the TCR [57,58,59]. By ablating the endogenous TCR followed by selective depletion of native TCR^+^ cells allows for the generation of highly potent, tumor specific CAR-T cells lacking any capacity to target non-CAR antigens [55,60,61]. In addition, “off-the-shelf” CAR-T cells are being developed using this technology to overcome the complex logistical issues of autologous CAR-T cell isolation, transduction, and infusion. Qasim et al. generated CAR-T (UCART19) cells from third party donors by lentiviral transduction of CAR-T and simultaneous transcription activator-like effector nuclease (TALEN)-mediated gene editing of the TCR α chain and CD52 loci [62]. These “off-the-shelf” CAR-T cells were then used to treat two infants with relapsed refractory ALL and bridge them to allogeneic stem cell transplantation. Currently, two phase 1 trials are ongoing each for the pediatric (NCT02808442) and the adult (NCT02746952) patients with ALL utilizing this approach.

Given the relative ease of multiplex strategies with CRISPR/Cas9 based editing, Ren et al. showed that it is feasible to combine lentiviral delivery of CAR and multiple genome editing of TCR, β2 microglobulin (β2M) and PD-1 simultaneously [55]. The triple gene disruption of TCR, β2M, and PD-1 yielded double-negative CD3 and HLA-I at 65% without any purification or selection, while a targeting efficiency of >90% at the protein level was routinely achieved for a single gene disruption. The TCR and HLA class I double-deficient T cells showed reduced alloreactivity. Finally, simultaneous triple genome editing by disruption of PD-1 led to enhanced in vivo antitumor activity of gene-disrupted CD19-specific CAR-T cells in a Nalm6-PD-L1 leukemia model. The same group developed a “one-shot” CRISPR protocol by incorporating multiple gRNAs into a CAR lentiviral vector [60]. With this approach, simultaneous, quadruple gene targeting of TCR, HLA-I, PD-1 and CTLA-4 was achieved. Based on these data, a phase I trial of NY-ESO-1 redirected CRISPR-edited T cells (NYCE cells) engineered to express NY-ESO-1 TCR and gene edited to eliminate endogenous TCR and PD-1 was submitted. These approaches are highly impactful; however, the generation of multiple simultaneous DNA breaks can lead to translocations and the ramifications of this are difficult to predict.

### 6.2. Knock-In Strategy of CAR into TRAC Locus

In opposition to mutagenic NHEJ-based strategies that are associated with mutagenic insertions and deletions, homology directed repair is an error free mechanism reliant on homologous repair templates that can be provided in trans. Recently, CRISPR/Cas9-directed genome editing has been reported for the specific targeting of a CAR gene into the TRAC locus [36]. High knockout (~70%) and knock-in (~40%) frequencies were reported with 95% of CAR positive cells being TCR negative. The ingenious design strategy placed the CAR gene under the control of the endogenous TRAC gene promoter. This resulted in uniform levels of CAR expression, averted tonic CAR-signaling, established effective internalization and re-expression of CAR following single or repeated exposure to antigen, and delayed effector T cell differentiation and exhaustion. While conventional CAR-T cells showed up to 50% positive expression of three markers of exhaustion (PD-1, LAG-3, and TIM-3) by day 17, TRAC-CAR showed less than 2% of these markers. This targeting of CAR-T to the TRAC locus resulted in T cells functioning like native T cells targeting tumor cells that outperformed the classical viral delivery-based CAR, showing superior anti-leukemic activity in a mouse. Given the potency of TRAC-CAR-T cells even at low doses, this strategy should enable faster and cheaper manufacturing. Further, targeted CAR gene integration might prove safer than the use of viral vectors and their potential genome-destabilizing insertional profile. 

## 7. Exploration of Immune Checkpoints Other than PD-1

A comparative analysis of gene expression in patients enrolled in ACT clinical trials showed that 156 genes related to immune function were differentially expressed [63]. Despite PD-1 being a promising candidate that is being pursued on multiple fronts, these data highlight the need to consider other immune regulators. Of these, LAG-3, TIM-3, CTLA-4, SHP-1, and adenosine 2A receptor (A2AR) have emerged as candidates for further enhancing the potency of immunotherapeutics. 

### 7.1. LAG-3

LAG-3, lymphocyte activation gene 3 also known as CD223, is an immunosuppressive molecule highly expressed on activated CD4 and CD8 T cells and Tregs [64]. Signaling occurs upon binding to MHC class II molecules, leading to the inhibition of effector functions and anergy. LAG-3 expression increases in CAR-T cells upon antigen engagement of target [36,38,39] and in the tumor microenvironment after CAR-T therapy [22]. Zhang et al. generated CD19 CAR-T cells with LAG-3 knockout using CRISPR-Cas9-mediated gene editing, with over 70% of efficiency [65]. However, no significant functional superiority of these cells over non-edited CAR-T cells was found. Given that LAG-3 and PD-1 are co-expressed on both CD4^+^ and CD8^+^ TILs and co-blockade of the LAG-3 and PD-1 pathways has been shown to improve anti-tumor CD8^+^ T cell responses [64,66], it warrants further investigation whether the dual blockade of PD-1 and LAG-3 in CAR-T cells can enhance the efficacy of CAR-T cells. 

### 7.2. TIM-3

TIM-3, a family member of T cell immunoglobulin and mucin domain proteins, is an inhibitory immune checkpoint molecule capable of suppressing type 1 T helper (Th1) responses and mediating T cell exhaustion [67]. In cancer patients, TIM-3 is upregulated on tumor antigen-specific CD8^+^ T cells and CD8^+^ TILs. Anti-TIM-3 monoclonal antibodies can increase pathways in parallel with T cell activation leading to the proliferation and cytokine production by tumor antigen-specific T cells [67,68]. Upregulation of TIM-3 may be a mechanism of adaptive tumor resistance to therapeutic PD-1 blockade [69] and combinatorial blockade of the PD-1 and TIM-3 pathways could reverse T cell exhaustion and restore anti-tumor immunity [70]. Similar to PD-1 and LAG-3, TIM-3 expression is also increased on activated CAR-T cells [36,38,39,71]. In a model of acute myeloid leukemia, there was a significant up-regulation of TIM-3 on CAR-T cells isolated from mice with relapsed disease compared with T cells isolated from mice in remission after CAR-T cell therapy [71]. Furthermore, the addition of systemic PD-1 or TIM-3 blockade to CAR-T cell treatment resulted in a synergistic anti-tumor activity suggesting TIM-3 blockade may useful in conjunction with CAR-T therapy. 

### 7.3. CTLA-4

CTLA-4, cytotoxic T lymphocyte antigen-4, is a central inhibitory immune checkpoint molecule expressed by activated T cells [72,73]. It has higher affinity for the costimulatory receptors CD80 and CD86 (B7-1 and B7-2) on antigen-presenting cells than the T cell costimulatory receptor CD28. After activation, T cells reduce expression of CD28 and increase expression of CTLA-4 leading to loss of costimulation through CD28. This results in cessation of proliferation and cytokine production. The anti-CTLA-4 antibody, ipilimumab, was the first immune checkpoint blockade to show promising results in cancer treatment; however, it can be associated with immune-related adverse events [73,74]. The role of CTLA-4 in CAR-T has been investigated with CD28 CD19-specific CAR-T cells that showed shRNA-mediated CTLA-4 down-regulation had no effect in the CAR-T cells. In contrast, CD19-specific CAR-T cells coexpressing CD80 but lacking costimulation domain showed significant increases in expansion and anti-tumor properties when CTLA-4 was knocked-down [74]. 

### 7.4. SHP-1

Src homology 2 domain-containing protein tyrosine phosphatase 1 (SHP-1) is an inhibitory protein tyrosine phosphatase that dephosphorylates TCR kinases such as Lck and ZAP70 and plays a role in the signal transduction of inhibitory checkpoint receptors [75]. In T cells, it is a negative regulator of antigen-dependent activation and proliferation [76]. Adoptive transfer of SHP-1 knockout T cells has been shown to be beneficial in a model of leukemia [77]. Moon et al. showed that CAR-TILs exhibit high expression of SHP-1, which rapidly declined 24 h after removal of the TILs from the tumors [39]. Addition of the SHP-1 inhibitor sodium stibogluconate significantly increased the killing ability of mesothelin-specific CAR-TILs and caused enhanced tumor-induced IFN-γ secretion. In this study a CAR with a dominant-negative SHP-1 (dnSHP-1) was employed and resulted in better control of PD-L1 expressing tumor growth compared to CAR-T cells alone [78]. In addition, CAR-T infiltration into the tumor was 3-fold higher in tumors harvested from mice that received dnSHP-1 expressing CAR-T cells. These data suggest that SHP-1 modulation may represent a novel way of blocking the suppression of CAR-T cells by multiple inhibitory pathways including PD-1.

### 7.5. Adenosine 2A Receptor

Adenosine is found at immunosuppressive concentrations within the tumor microenvironment and can be generated from extracellular ATP in a stepwise manner by the ectoenzymes CD39 and CD73 in tumor cells or innate cells such as Tregs or MDSCs [79,80]. Adenosine is thought to predominantly suppress endogenous antitumor T cell responses through the stimulation of A2ARs expressed on activated T cells. Beavis et al. explored the possibility that targeting A2ARs could enhance CAR-T cell activity [81]. They demonstrated that CAR-T cells upregulate A2ARs upon antigen-specific stimulation in vitro and in vivo. Consequently, A2AR-deficient CAR-T cells exhibited significantly greater therapeutic efficacy than wild-type CAR-T cells. Moreover, they observed that pharmacologic blockade of A2ARs can enhance the efficacy of CAR-T cell responses. In particular, augmentation of CAR-T efficacy was pronounced when A2AR and PD-1 blockade was combined. This study highlights how the combinatorial immune-checkpoint blockade can act synergistically to augment the efficacy of CAR-T cells.

## 8. Summary and Future Directions

The combinatorial approach(es) of CAR-T and immune-checkpoint blockade has shown tremendous potential in multiple preclinical models and is being applied clinically. This area of investigational and clinical pursuit is synergistic in its application and design. CAR-T cells have achieved some stunning therapeutic successes as a standalone therapy and their on-target tumor reactivity has been and will continue to be augmented by immune checkpoint blockade. To date PD-1 has been a lead candidate and modulatory strategies have included antibody blockade, dominant negative, CSR, shRNA, and genome engineering. The greatest benefits may be achieved by a combined checkpoint blockade of multiple inhibitory pathways (e.g., PD-1, CTLA-4, LAG-3). Further, systemic comparative analysis of gene expression for inhibitory pathway mediators in the tumor setting will provide further understanding of the exhaustion mechanism(s) tumors employ and will reveal new targets of opportunity [63]. Ultimately, the ideal combination of CAR-T with concurrent or adjuvant coinhibitory blockade will enhance CAR-T cell potency. In order to achieve this promise, the immunotherapy field stands at the forefront of integrating multiple disciplines—big data analysis, gene and cell engineering, rational antibody design, and immune network discovery and definition-for the coordinated effort of achieving maximal anti-tumor responses with minimal off-tumor collateral toxicity.

## Figures and Tables

**Table 1:** Open clinical trials exploring the role of immune-checkpoint blockade with CAR-T cell therapy.

Clinical Trials.gov Identifier (ref.)	CAR-T (Trial Nickname)	Blockade of PD-1 Axis	Target Disease	Sponsor
NCT02706405	JCAR014	Durvalumab; on D28 + up to 10 doses, every 4 weeks or on D-1 and -28 (and additional dose allowed)	DLBCL, double-hit lymphoma, PMBL, tDLBCL	Fred Hutchinson Cancer Research Center
NCT03310619	JCAR017 (PLATFORM)	Durvalumab; Dose/schedule: NA	Aggressive B-NHL	Celgene
NCT02926833 [48]	KTE-C19 (ZUMA-6)	Atezolizumab; 4 doses every 3 weeks from D1, D14 or D21 in each cohort	DLBCL	Kite pharma
NCT03287817	CD19/22 CAR-T (ALEXANDER)	Pembrolizumab as consolidation Dose/schedule: NA	DLBCL	Autolus limited
NCT02650999 *	CTL019	Pembrolizumab; every 3 weeks up to 18 doses	CD19^+^ DLBCL, FL, MCL	University of Pennsylvania

Abbreviations: B-NHL, B-cell non-Hodgkin lymphoma; DLBCL, diffuse large B-cell lymphoma; FL, follicular lymphoma; MCL, mantle cell lymphoma; PMBL, primary mediastinal large B-cell lymphoma; tDLBCL, transformed diffuse large B-cell lymphoma. * Phase I/II study of pembrolizumab in patients failing after CD19-specific CAR-T cell therapy for relapsed or refractory CD19^+^ non-Hodgkin lymphoma. The trial does not aim to study the combination of CAR-T and PD-1 blockade but the role of salvage therapy of pembrolizumab in patients whose disease progressed after CTL019.

**Table 2:** Clinical trials involving CAR-T cells harnessing immune checkpoint blockade.

Clinical Trials.gov Identifier	Title or CAR-T Strategy	Target Disease	Sponsor
NCT03179007	MUC1-specific CAR-T cells producing CTLA-4 and PD-1 antibodies	MUC1^+^ solid tumors	Shanghai Cell Therapy Research Institute
NCT03182816	EGFR-specific CAR-T cells producing CTLA-4 and PD-1 antibodies	EGFR^+^ solid tumors	Shanghai Cell Therapy Research Institute
NCT03182803	Mesothelin-specific CAR-T cells producing CTLA-4 and PD-1 antibodies	Mesothelin^+^ solid tumors	Shanghai Cell Therapy Research Institute
NCT03030001	Mesothelin-specific CAR-T cells producing PD-1 antibodies	Mesothelin^+^ solid tumors	Ningbo Cancer Hospital
NCT02873390	EGFR-specific CAR-T cells producing PD-1 antibodies	EGFR family^+^ solid tumors	Ningbo Cancer Hospital
NCT02862028	EGFR-specific CAR-T cells producing PD-1 antibodies	EGFR family^+^ lung, liver or stomach cancer	Shanghai International Medical Center
NCT03170141	EGFRvIII-specific CAR-T cells producing PD-1 and PD-L1 antibodies	Glioblastoma Multiforme	Shenzhen Geno-Immune Medical Institute
NCT03298828	CD19-specific CAR-T; PD-1 knockout	CD19^+^ B-cell leukemia or lymphoma	Third Military Medical University
NCT03208556	CD19-specific CAR-T; PD-1 shRNA	CD19^+^ B-cell lymphoma	Peking University

Abbreviation: EGFR, epidermal growth factor receptor.

## Supplementary Materials

Supplementary materials can be found at http://www.mdpi.com/1422-0067/19/2/340/s1.
