# Supplementary material for: Incorporation of Immune Checkpoint Blockade into Chimeric Antigen Receptor T Cells (CAR-Ts): Combination or Built-In CAR-T
Source: Int J Mol Sci. 2018 Jan 24;19(2):340. doi: 10.3390/ijms19020340 (PMC5855562; doi:10.3390/ijms19020340)
Supplement: Supplementary file 1 [file ijms-19-00340-s001.pdf]

International Journal of *Molecular Sciences*

MDPI

Supplementary table 1. Summary of preclinical studies on the combination of immune checkpoint blockade and CAR-T cells

Ref	Mode of checkpoint blockade	CAR structure (target-costimulatory domain)	Experimental model	Key findings
John 2013 [42]	Anti-PD-1 Ab<br>250 μg on D0, 3, and 7	HER2-CD28 CAR	Mouse sarcoma cells, 24JK-Her2<br>Mouse breast carcinoma cells, e0771-Her2	Increased PD-1 expression on CAR-T following antigen stimulation<br>A significant improvement in tumor growth inhibition by the combo of CAR-T and anti-PD-1 antibody with increased CAR-T function
Cherkassky 2016 [38]	Anti-PD-1 Ab, 10 mg/kg, ip 3 times or continuously every 5 days from D0 to D85,<br>PD-1 DNR coexpression or<br>PD-1 shRNA blockade	Mesothelin-CD28 CAR<br>Mesothelin-41BB CAR	Human pleural mesothelioma cells, MSTO-211H (orthotopic pleural mesothelioma model)<br>Intrapleural CAR-T injection	Immune exhaustion of CAR-T following in vivo antigen exposure<br>Delayed exhaustion of 41BB CAR-T cells than CD28 CAR-T cells<br>Inhibition of CAR-T cell effector functions by tumor-cell PD-L1<br>Rescue of CD28 CAR-T cell function in vivo by PD-1 antibody or PD-1 DNR expression and in vitro by PD-1 targeting shRNA expression
Moon 2014 [39]	Ex-vivo blockade of PD-1 with 10 μg/mL anti-PDL1 Ab or<br>SSB, 25 μg/mL for SHP-1 inhibition	Mesothelin-41BB CAR	Human mesothelioma cell line, EMMESO	Hypofunction of CAR TILs with upregulation of SHP-1, PD-1, LAG-3, TIM-3 and 2B4, which was reversible.<br>Enhanced CAR-T cells ex vivo killing function by blockade of PD-1 or SHP-1
Moon 2016 [43]	Anti-PD-1 Ab, 10 mg/kg every 5 days ip	NY-ESO1–reactive Ly95 TCR T-cells	Human lung cancer cell line, A549-A2-ESO	Reversible hypofunction of Ly95 TILs with high expression of PD-1, LAG-3 and TIM-3. Augmentation of Ly95 T cells' efficacy in vivo by anti-PD-1 antibody
Burga 2015 [44]	Anti-PD-L1 Ab, 10 μg ip on D2, 4, 6, 8, 10, 12, 14, and 16 post-tumor injection	CEA-CD28 CAR	Murine colon adenocarcinoma cell line, MC38-CEA	Suppression of CAR-T through STAT3-dependent PD-L1 expression by MDSC<br>Rescue of CAR-T efficacy by MDSC depletion, GM-CSF neutralization or PD-L1 blockade
Suarez 2016 [49]	Bicistronic CAR secreting human anti-PD-L1 Ab	CAIX-CD28 CAR	Orthotopic model of human ccRCC cell lines, skrc52 and skrc59	Reversal of CAR-T exhaustion in anti-PD-L1 Ab secreting CAR-T with decreased expression of PD-1, TIM-3 and LAG-3 and better tumor control
Li 2017 [50]	Bicistronic CAR secreting human anti-PD-1 Ab vs. systemic anti–PD-1 Ab, 125 μg per mouse on days 1, 5, 9 and 12 after T cell transfer	CD19-CD28 CAR	Human lung cancer cell, NCI H292-CD19<br>Human ovarian cancer cell, SKOV3-CD19	Enhanced antitumor activity of anti-PD-1 secreting CAR-T cells with lower expression of PD-1 and LAG-3
Tanoue [51]	Helper-dependent adenovirus (HDAd) that express a PD-L1 blocking mini-antibody or anti-PD-L1 IgG (100 μg ip at day 0, 3, and 6)	HER2-CD28-CAR	Human prostate cancer cell line PC-3, human NSLC cell line A549, human HCC cell line HepG2, human SqCC line SiHa	Superiority of local production of PD-L1 mini-body by CAd-VECPDL1 combined with administration of CAR-T cells

Liu [53]	CSR of PD-1 and CD28<br>Anti-PD-1 Ab (pembrolizumab ip, 10 mg/kg every 5 days)<br>PD-1 DNR	CD19-41BB-CAR<br>Mesothelin-41BB-CAR<br>PSCA-41BB-CAR	EMMESO; Human leukemic cells, PD-L1+ Nalm6; Human CML cell line, CD19+ PD-L1+ K562; Human prostate cancer cell line, PSCA+ PD-L1+ PC3	CAR T cells with CSR of PD1:CD28 enhanced CAR infiltration to tumor, decreased susceptibility to tumor-induced hypofunction, and attenuation of inhibitory receptor expression compared with treatments with CAR T cells alone or PD-1 antibodies.
Rupp [54]	CRISPR/Cas9 mediated disruption of PD-1	CD19-41BB-CAR	CD19+ PD-L1+ K562	Cas9RNP-mediated PD-1 disruption augmented CAR-T cell mediated killing of tumor cells in vitro and enhanced clearance of PDL1+ tumor xenografts in vivo.
Ren [55]	CRISPR/Cas9 mediated disruption of TRAC/TRBC/B2M/PD-1	CD19-41BB-CAR<br>PSCA-41BB-CAR	PDL1+ Nalm6<br>PSCA+ PD-L1+ PC3	Multiple deliveries of gRNAs disrupt genes in human primary T cells with high efficiency without impairing effector function.<br>Reduced alloreactivity of TCR and B2M double-disrupted T cells<br>Disruption of PD-1 in CAR T cells leads to enhanced antitumor efficacy
Ren [60]	CRISPR/Cas9 mediated disruption of TRAC/TRBC/B2M/Fas/PD-1/CTLA-4	CD19-41BB-CAR<br>PSCA-41BB-CAR	CD19+ K562<br>Nalm6	Efficient multiplex genome editing by a one-shot CRISPR protocol by incorporating multiple gRNAs in a CAR lentiviral vector
Eyquem [36]	Knock-in CD19 CAR-T to TRAC locus by CRISPR/Cas9	CD19-CD28-CAR	Nalm6	Knock-in of CAR-T to TRAC locus results in uniform CAR-T expression enhances CAR-T potency by preventing exhaustion.
Liu [65]	CRISPR/Cas9 mediated disruption of LAG-3	CD19-NA-CAR	CD19+ K562<br>Raji	Ca9RNP-mediated LAG-3 disruption did not increase activity of CAR-T compared with stand alone CAR-T
Kenderian [71]	Anti-PD-1 Ab on D3, 6, 9, 12 (10 mg/kg/dose)<br>Anti-TIM-3 Ab on D3, 6, 9, 12 (10 mg/kg/dose)	CD123-41BB-CAR	AML cell line, MOLM	PD-1 and TIM-3 pathways are involved in CAR-T exhaustion and dysfunction in AML model.
Condomines [74]	CTLA-4 shRNA	CD19-CD28-CAR	NALM6	CTLA-4 down-regulation increases efficiency of CD19 CAR-T-cell expressing CD80 but not that of CD19-CD28 CAR-T cells
Moon [78]	Bicistronic CAR expressing dnSHP-1<br>SSB, 20 mg/kg i.m every 2 days	Mesothelin-41BB CAR	EMMESO<br>PDL1+ EMMESO	Higher infiltration into tumor and better control of tumor by CAR-T/dnSHP-1
Beavis 2017 [81]	A2AR knock-down by shRNA<br>Anti-PD-1 Ab (200 µg, D0 and 4, 8)<br>SCH58261 1 mg/kg, daily<br>ZM241385 1 mg/kg daily	Murine anti-human HER2-CD28 CAR<br>Human HER2-CD28 CAR	Mouse sarcoma cells, 24JK-Her2<br>Mouse breast carcinoma cells, e0771-Her2<br>Primary melanoma cell line	Increased A2AR expression and suppression of CAR-T cells after CAR activation, which was reversible upon pharmacological or genetic targeting of A2AR<br>Increased CAR-T activity upon dual blockade of anti-PD-1 and A2AR

Abbreviations: A2AR, adenosine 2A receptor; AML, acute myeloid leukemia; ccRCC, clear cell renal cell carcinoma; B2M, beta-2-microglobulin; CSR, chimeric switch receptor; DNR, dominant negative receptor; GM-CSF, granulocyte-macrophage colony-stimulating factor; MDSC, myeloid-derived suppressor cells; NA, not available; RNP, ribonucleprotein ; SSB, Sodium stibogluconate; TIL, tumor-infiltrating lymphocyte; TRAC, TCR α chain constant region; TRBC, TCR β chain constant region; * SCH58261 and ZM241385 are A2AR antagonists.
